# The role of GnRH metabolite, GnRH-(1-5), in endometrial cancer

**DOI:** 10.3389/fendo.2023.1183278

**Published:** 2023-04-14

**Authors:** Madelaine J. Cho-Clark, Allison Watkins, T. John Wu

**Affiliations:** Department of Gynecologic Surgery & Obstetrics, Uniformed Services University of the Health Sciences, Bethesda, MD, United States

**Keywords:** GnRH-(1-5), endometrial cancer, GnRH, GPR101, EP24.15

## Abstract

From the time of its discovery and isolation in the mammalian hypothalamus, the decapeptide, gonadotropin-releasing hormone (GnRH), has also been found to be expressed in non-hypothalamic tissues and can elicit a diverse array of functions both in the brain and periphery. In cancer, past studies have targeted the gonadotropin-releasing hormone receptors (GnRHR) as a way to treat reproductive cancers due to its anti-tumorigenic effects. On the contrary, its metabolite, GnRH-(1-5), behaves divergently from its parental peptide through putative orphan G-protein coupled receptor (oGPCR), GPR101. In this review, we will focus on the potential roles of GnRH-(1-5) in the periphery with an emphasis on its effects on endometrial cancer progression.

## Introduction

The gonadotropin-releasing hormone (GnRH) is a central regulator of mammalian reproductive function through the hypothalamic–pituitary–gonadal (HPG) axis. Since its discovery in the porcine hypothalamus, over 20 different primary structures of GnRH and its receptors have been identified and studied across various species (1–4). The first form of GnRH in vertebrates (pGlu-His-Trp-Ser-Tyr-Gly-Leu-Arg-Pro-Gly-NH_2_) is designated as GnRH-I, contains highly conserved sequences on NH_2_ and COOH terminal residues, pGlu-His-Trp-Ser and Pro-Gly respectively (4). While GnRH regulation of the HPG axis have been extensively studied, its role in gonadotrope desensitization or fertility reestablishment in hypogonadal patients has made GnRH analogues and antagonists an appealing target for a wide variety of clinical treatments (5–12). Furthermore, a growing number of studies have determined that GnRHR stimulation by GnRH analogs induces antiproliferative and antimetastatic effects in various types of tumors, therefore, presenting GnRHRs as a good candidate for therapeutic intervention for cancers (4, 13–15).

While GnRH and its pharmacologic analogs impede cancer progression, its metabolite, GnRH-(1–5), elicits an opposing response. Our previous studies have demonstrated that GnRH-(1–5) is a biologically active pentapeptide and its mechanism of action is autonomous from its parental peptide (16). In this review, we will describe the metabolism of GnRH to generate GnRH-(1–5), provide a brief overview on the discovery of its bioactivity, and discuss how the biological responses elicited by GnRH-(1–5) in endometrial cancer cells are mediated by GPR101 to stimulate cell growth and metastatic behavior. Our studies suggests that GnRH-(1–5) is more than a metabolic byproduct and targeting its putative receptors may offer a therapeutic target for halting cancer progression.

## GnRH is metabolized to generate bioactive GnRH-(1–5)

The decapeptide, GnRH, is metabolized by zinc metalloendopeptidase EC 3.4.24.15 (EC 3.4.24.15 designation based on the International Union of Biochemistry and Molecular Biology (IUBMB) Enzyme Nomenclature; abbreviated as EP24.15 or THOP1), to generate the pentapeptide GnRH-(1–5) (17). This thimet oligopeptidase belongs to a class of zinc dependent metalloendopeptidases expressed in mammalian tissues; most notably in the brain, pituitary, and testis (18–20). First identified as a 75kDa neuropeptide peptidase in the soluble fraction of rat brain homogenates, it was originally thought to be predominantly localized in the cytoplasm. However, EP24.15 has subsequently been found temporally localized to the extracellular surface of the plasma membrane, as well as synaptosomes and the exofacial leaflet of the lipid raft microenvironment (21–25).

Historically, this peptidase was recognized for its sole function as a classic degrading enzyme terminating its substrate bioactivity by reducing or eliminating the binding potential to its cognate receptor. However, EP24.15 has since been recognized as possessing other abilities to regulate cellular activities. A previous study has demonstrated that within the purified synaptosomal membrane of the rat brain, EP24.15 was able to transform neoendorphin into Leu-enkephalin; an example of transforming an inert precursor into an active neuropeptide (25). EP24.15 can also modulate a peptide to alter its bioactivity in opposition of its original physiological effect, as exhibited by altering angiotensin-I (Ang-I) into fragment angiotensin-(1–7) (Ang-(1–7)) (26–28). Furthermore, our lab has demonstrated that EP24.15 can convert bioactive GnRH-I into another bioactive pentapeptide, GnRH-(1–5), to elicit lordosis behavior in female rats through a receptor independent of its parental receptor, GnRHR-I (16).

The manner of specificity in which GnRH is metabolized suggests that EP24.15 converts rather than simply generating a degraded peptide as an inactive final byproduct. Furthermore, EP24.15 displays preferential cleaving of hydrophobic residues at positions P1, P2 and P30 in larger peptides and P10 in smaller peptides; denoting this fragmentation as a highly distinctive process (29). *In vitro*, the hydrolysis of GnRH involves a two-step mechanism requiring zinc as a cofactor bound to the active site with the classic HEXXH motif, thiol activation, and phosphorylation of serine residue 644 (Ser644) by protein kinase A (PKA) (18, 30–33).

Kinetic studies in ovine hypothalamic extracts indicated that EP24.15 phosphorylation decreases its affinity for the α-amidated decapeptide GnRH (32, 33). However, upon cleavage of glycine (Gly^10^) at the carboxyl terminal by prolyl endopeptidase (PE), the intermediate fragment, GnRH-(1–9) becomes a greater substrate for EP24.15 by 10-15-fold (33). This study suggests that basic structural conditions are required for efficient and successful interaction with the binding site and that the amidation of the carboxyl terminal is necessary to avert any nonspecific degradation of GnRH (21). Subsequently in the second step, EP24.15 rapidly cleaves GnRH-(1–9) at the Tyr^5^-Gly^6^ bond to generate biologically active product, GnRH-(1–5) (32, 33).

## The discovery of GnRH-(1–5) biological activity

The widely accepted paradigm that a cleaved peptide becomes biologically inactive has been disputed by a growing body of literature that suggests otherwise (34). These fragmented peptides appear to regulate a diverse array of functions that are autonomous of its parental peptide (32, 35). In relation to the focus of our review, previous researchers have speculated that GnRH metabolite, GnRH-(1–5), may be a biologically active fragment and warrants further investigations (16, 34, 36–39).

One of the first observations of possible bioactivity were seen in the hypothalamic rat explants where treatment with GnRH-(1–5) resulted in the reversible suppression of the spontaneous pulsatile secretion of GnRH. This suppression was similar in manner to the NMDA competitive antagonist, AP-5 (36). This study suggests that GnRH-(1–5) may act through NMDA receptors to mediate an inhibitory autofeedback of GnRH secretion in various types of cells. Our previous expression study in the immortalized mouse neuron GT1-7 cell line demonstrated that treatment with GnRH-(1–5) stimulated GnRH-I mRNA expression in contrast to GnRH-I treatment, which demonstrated a negative autoregulatory feedback effect on GnRH-I expression (37). Interestingly, the effect of GnRH-(1–5) on LH release may be mediated by kisspeptin neurons (39). In the periphery, our studies with the human Ishikawa cell line, a commonly used model for GnRH-I effects on endometrial cancer studies, demonstrated that GnRH-(1–5) had no effect on the gene expression of the GnRH-I system unlike its parental peptide (38).

In addition to regulating gene expression, GnRH-(1–5) can also regulate lordosis behavior in estradiol-primed ovariectomized female rats (16). The intracerebroventricular (ICV) administration of GnRH-(1–5) elicited lordosis, indicating that the metabolite was just as effective in stimulating sexual behavior as its parental peptide, GnRH-I. This observation was further validated through the use of immunoneutralization studies with antibodies to EP24.15, in which GnRH-I facilitated lordosis was inhibited whereas the GnRH-(1–5)-facilitated lordosis remained unaffected (16). Collectively, these studies indicate that GnRH-(1–5) has different effects from its parental peptide, and its mechanism of action may be mediated through receptors independent of GnRHR (**Table 1**).

**Table 1:** Articles investigating GnRH-(1–5) biological activity in the brain and periphery.

Tissue/Behavior	Functional Effects	Reference
Hypothalamic rat explants	Reversible suppression of spontaneous pulsatile GnRH secretion	(36)
GT1-7 immortalized mouse neuron cell line	Stimulated increase in GnRH-I mRNA expression	(37)
Kisspeptin neurons in ovariectomized and estrogen-treated Wistar-Imamichi female rats	Increase in plasma LH concentration	(39)
Endometrial cancer cell line: Ishikawa	Stimulated increase in GnRH-II and GnRHR-II mRNA expression	(38)
Estradiol primed ovariectomized female rats	Elicited the lordosis response	(28)
Endometrial cancer cell line: Ishikawa	Increased cell proliferation, suppressed caspase-3/7 activity, and downregulated ERK1/2 expression	(40)
Endometrial cancer cell line: Ishikawa	Stimulated EGF release, increased phosphorylation of EGFR, promoted cell migration, and identified GPR101 as putative receptor	(41)
Endometrial cancer cell line: Ishikawa and ECC-1	Increased MMP-9 activity to stimulate EGF release, enhanced cell migration and invasion	(42)

## Identification of GPR101 as a putative GnRH-(1–5) receptor

Putative GnRH-(1–5) receptors were discovered by utilizing a high-throughput β-arrestin recruitment assay (43). One of the receptors identified for positive binding was orphan G protein-coupled receptor 101 (GPR101). A previous study noted that the promoter region of the GPR101 gene was hypermethylated in colorectal cancer, therefore, future studies were focused on GPR101 as a possible receptor for GnRH-(1–5) (44). The relationship between GnRH-(1–5) to GPR101 was validated through siRNA mediated downregulation of GPR101 expression studies and the reversal of GnRH-(1–5) effects on EGFR phosphorylation and migration (45). These findings implicate GPR101 as the receptor GnRH-(1–5) acts on to transactivate EGFR phosphorylation in the Ishikawa cell line.

The GPR101 gene is located on chromosome X (Xq26.3) which encodes for a 508-aa, Class A (rhodopsin-like) GPCR protein. It is highly expressed in the fetal pituitary during the somatotrope maturation process as well as the hypothalamus, nucleus accumbens, and other tissues (45–49). While it shares ~30% sequence homology in the transmembrane regions with GPCRs RE2, α-1A-adrenergic receptor, and the serotonin 5HT1A receptor, early studies utilizing the knowledge-restricted hidden Markov model-based algorithm and determination of cAMP levels in overexpressing human GPR101 in human embryonic kidney (HEK293) or rat pituitary GH3 cells indicates that GPR101 may be constitutively coupled to G_αs_ signaling pathway (46, 50, 51). Other studies utilizing the chinese hamster ovary (CHO-K1) or the Ishikawa cell line report that GPR101 does not constitutively activate or stimulate the cAMP pathway but suggests possible coupling to other G protein mediated pathways such as the G_αi_ or G_αq/11_ pathway (52, 53). These observations suggest that GPR101 and its G-protein selectivity may be tissue dependent and warrants further investigations in future studies. Although studies thus far have focused primarily on GPR101 and its role in endocrine related disorders such as X-linked acrogigantism (X-LAG) (54, 55), new emerging studies indicate other possible functions in mediating the hypothalamic control of energy homeostasis, pro-resolving actions in leukocytes to control inflammation, and stimulating cell proliferation and metastatic behavior in endometrial cancer cells (45, 48, 49, 56, 57).

## GnRH-(1–5) effects on cell proliferation in endometrial cancer cells

As stated previously, GnRH-I and its analogs offer treatment to a wide array of conditions including sex hormone-dependent diseases seen in prostate and gynecological cancers (12, 19, 58–63). The activity of GnRH is regulated through GnRHR *via* distinctive signal transduction pathways in a tissue-dependent manner. Most studies associate the antitumorigenic activity of GnRHR through coupling with the Gα_i_ pathway. This pathway is correlated to the activation of apoptotic signaling cascades, augmentation of phosphotyrosine-phosphatases, promotion of cell cycle arrest, and impeding the MAPK signaling pathway (40–42, 64–66). However, some studies from cells in other tissues have observed GnRH-I coupled to the Gα_q_ subunit (67, 68).

While GnRH analogs are known to have antitumorigenic effects, in our studies, GnRH-(1–5) exerts an opposing effect by promoting cell proliferation and invasion in endometrial cell lines. Our initial study investigated whether a wide range of dosage treatments with GnRH vs GnRH-(1–5) can affect cell proliferation and alter markers for MAPK signaling pathway in the Ishikawa cell line (69). After 24h of treatment, this study demonstrated that GnRH and GnRH-(1–5) had diverging effects on cell proliferation; GnRH decreased cell proliferation whereas GnRH-(1–5) increased cell proliferation. Furthermore, GnRH-(1–5) suppressed caspase-3/7 activity and downregulated ERK-1/2 expression, suggesting for the first time that cell growth and proliferation may be linked to an apoptotic process in endometrial cancer cells.

## GnRH-(1-5) signaling pathway

**Figure 1:**
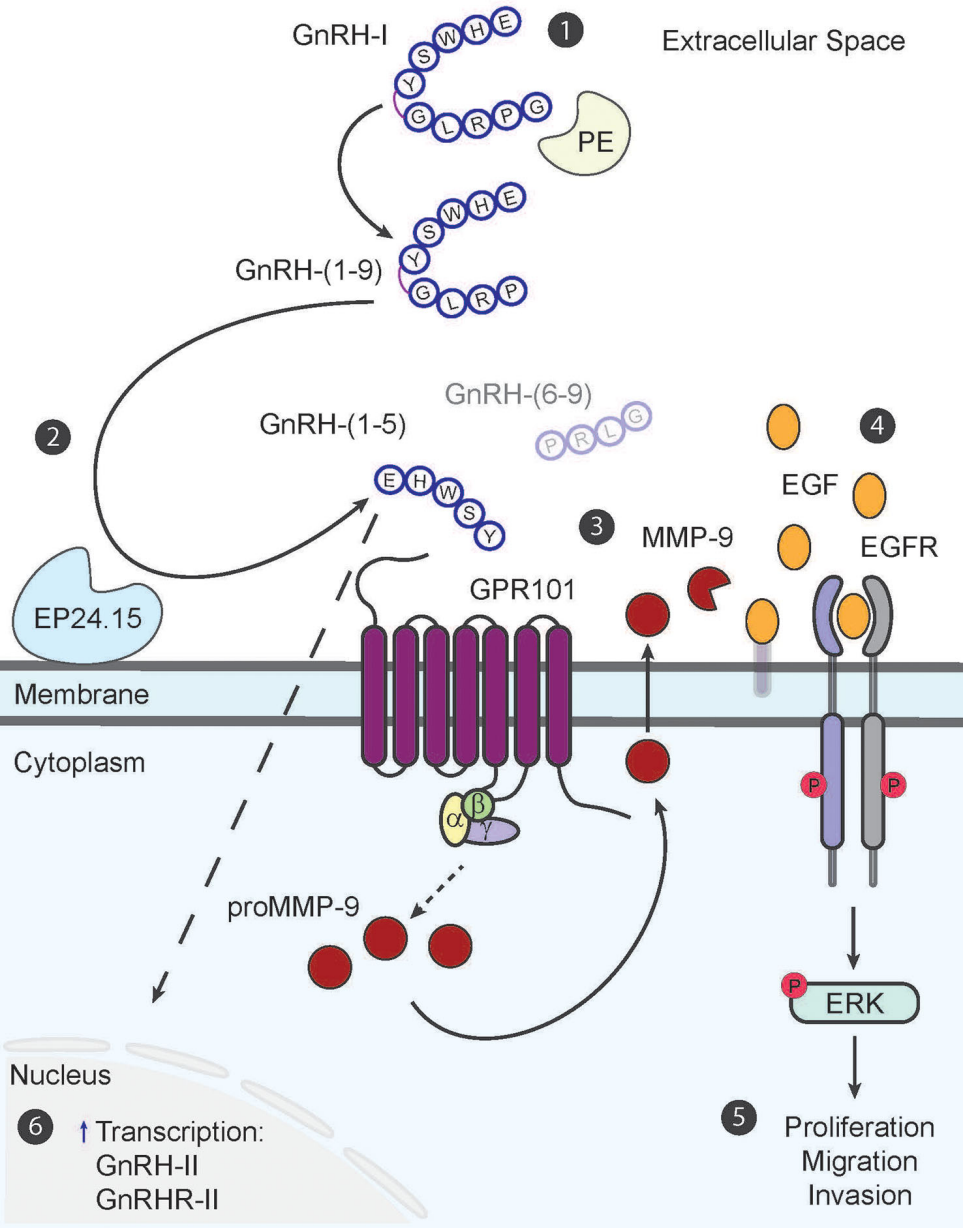
The GnRH-(1–5) paradigm in endometrial cancer cells. The metabolite GnRH-(1–5) is generated in a two-step process from decapeptide, GnRH-I (1). The first step is the removal of Gly10 at the C-terminus by enzyme, prolyl endopeptidase (PE), to generate intermediary fragment, GnRH-(1–9) (2). The second step involves the hydrolyses of GnRH-(1–9) by enzyme, EP24.15, at the covalent bond between Tyr5–Gly6 to produce bioactive pentapeptide fragment, GnRH-(1–5) and tripeptide GnRH-(6–9) (3). GnRH-(1–5) in turn binds to GPR101 to stimulate MMP-9 activity to increase EGF release into the extracellular space in a G-protein dependent manner (4). The release of EGF subsequently increases EGFR phosphorylation at three tyrosine sites (992, 1045, 1068) and initiates signaling cascades (5). Downstream phosphorylation of ERK results in increased cell proliferation, migration, and invasion; all important factors for inciting metastatic potential (6). GnRH-(1–5) can also regulate GnRH-II and GnRHR-II gene expression; however this mechanism still remains elusive.

The EGFR signaling pathway is heavily implicated in its role in cancer progression (70, 71). Dysregulation of this signaling pathway in endometrial cancer can lead to transforming tumors into a more aggressive metastatic phenotype; therefore, we performed a study to determine whether GnRH-(1–5) can alter the EGFR signaling pathway in the endometrial cancer Ishikawa cell line (72–75). Our studies suggest that GnRH-(1–5) stimulates epidermal growth factor (EGF) release, increases phosphorylation of EGFR at three tyrosine sites (992, 1045, 1068), and promotes cell migration (**Figure 1**) (45); however, GnRH and GnRH analogs, (D-Ser^6^)-GnRH or (D-Trp^6^)-GnRH had no effect on EGFR phosphorylation and impeded cellular migration compared to untreated cells. Pre-incubation with GnRHR antagonist, Antide, had no effect on GnRH-(1–5) ability to stimulate EGF release and EGFR phosphorylation demonstrating a novel mechanism that’s independent of GnRHR in mediating these effects. Furthermore, studies with G-protein antagonist peptide (GPAnt-2) suggested effects observed with GnRH-(1–5) were G-protein dependent.

Our most recent study with GnRH-(1–5) has determined that its effects are dependent on increased matrix metallopeptidase-9 (MMP-9) enzymatic activity in two different endometrial cancer cell models, the Ishikawa and ECC-1 cell lines (57). Upon binding to GPR101, GnRH-(1–5) increases MMP-9 activity to augment EGF release. This in turn increases EGFR phosphorylation to stimulate cellular migration and invasion. These results indicate the important physiological relevance of GnRH-(1–5) effects on mediating MMP-9 activity in increasing the metastatic potential of endometrial cancer cells.

## Summary

The novelty of GnRH-(1–5) and its role in the pathophysiology of endometrial cancer adds another layer of complexity to our current understanding of the GnRH paradigm in endometrial cancer. Future studies with GnRH-(1–5) should investigate its effects on other growth factors and related signaling pathways highly implicated in endometrial cancer, such as transforming growth factor beta (TGFβ), vascular endothelial growth factor (VEGF), and platelet-derived growth factor (PDGF) (76–80). The TGFβ pathway is a well-known contributor to the malignant transformation of the precursor lesion endometrial intraepithelial neoplasm (EIN) into invasive carcinoma and rising levels of VEGF are linked to the development of endometrial cancer (76). The presence of PDGF in the microenvironment of carcinomatous endometrium is a hallmark in cancer-associated fibroblasts. PDGF is shown to promote immune cell recruitment through the modulation of the PI3K/Akt and MAPK/ERK pathway to stimulate endometrial cancer cell proliferation, which we have already identified to be a downstream effect of the GnRH-(1–5) signaling pathway (45, 57, 76).

In addition to investigating GnRH-(1–5) effects on other growth factors, exploration of GnRH-(1–5) signaling through another putative receptor, GPR173, should be considered since our studies in the brain have determined that GnRH-(1–5) can act on GPR173 to inhibit neuronal migration (81). Upon stimulation by GnRH-(1–5), GPR173 recruits β-arrestin and phosphatase and tensin homolog (PTEN) as adaptor proteins to inhibit the phosphorylation of signal transducer and activator of transcription 3 (STAT3) leading to decreased migration (43, 81). In women with endometriosis, reduced PNX levels and GPR173 expression may be responsible for HPG axis dysregulation (82).

These new insights may contribute to a better understanding of the pathophysiology of endometrial cancer and provide the basis for a new strategy for diagnosis. Furthermore, in our comparative transcriptome analysis between patient and endometrial cancer cell lines, we identified that one of the top five signaling pathways involved in cancer progression is the neuroprotective role for THOP1 in Alzheimer’s disease; shift from being up- to downregulated as approaching advanced cancer stage III (83). The THOP1 gene encodes for the protein EP24.15, which we have described previously, as the primary enzyme that metabolizes GnRH to GnRH-(1–5). Future studies should address the relationship between increased EP24.15 expression and enzymatic activity to generate GnRH-(1–5) to all its related markers identified to ascertain its role in driving cancer progression since a prior study has implicated augmented EP24.15 activity in prostate cancer (29).

Importantly, continuing to understand the effects of GnRH-(1–5) on endometrial cancer progression will provide future targets for pharmaceutical intervention. Currently, the mainstay treatment for early-stage disease is surgery however depending on the stage of disease and other risk factors, adjuvant radiotherapy and/or chemotherapy is used. Given that primary management for endometrial cancer is surgical intervention, the discovery of pharmaceutical targets will provide the most benefit for patients with comorbidities who are unsuitable for surgery and pre-menopausal or hormone insensitive patients who want to preserve their fertility.

## Author contributions

MC-C and AW drafted and edited the manuscript. MC-C generated the Table and Figure. TW edited the drafts and edited the manuscript. All authors contributed to the article and approved the submitted version.
